# Efficiency gains resulting from the ordinal analysis of a functional outcome scale: a case study of a major phase III stroke trial

**DOI:** 10.1186/1745-6215-12-S1-A67

**Published:** 2011-12-13

**Authors:** Gordon D Murray, Else Charlotte Sandset, Philip MW Bath, Eivind Berge

**Affiliations:** 1 Edinburgh MRC Hub for Trials Methodology Research, University of Edinburgh, Edinburgh, EH8 9AG, UK; 2 Department of Internal Medicine, Oslo University Hospital Ullevål, Oslo, NO-0407, Norway; 3 Stroke Trials Unit, University of Nottingham, Nottingham, NG5 1PB, UK

## Background

Phase III clinical trials in areas including acute stroke and traumatic brain injury commonly use ordinal functional outcome scales as their primary outcome measure. Conventionally these scales are analysed by dichotomising the ordinal scale into a binary scale:- ‘dead or dependent’ versus ‘independent’. This can potentially discard much relevant information, reducing both the clinical relevance of the results and the statistical efficiency of the analysis.

## Methods

Methodological work in stroke (by the OAST Group) and in traumatic brain injury (by the IMPACT Investigators) has demonstrated that using more appropriate approaches to the analysis of ordinal outcome scales, such as proportional odds regression or the ‘sliding dichotomy’, can potentially lead to substantial efficiency gains relative to the conventional dichotomous analysis. However, to date relatively few trials have prospectively adopted ordinal techniques for their primary analysis. We report here how in SCAST [1], a major Phase III trial of blood pressure reduction in acute stroke, ordinal methods were adopted for the primary analysis of the modified Rankin Scale (mRS), an ordinal functional outcome scale.

## Results

Since ordinal methodology was evolving in parallel with the conduct of the trial, the Statistical Analysis Plan was not finalised until close to database lock. It was decided to use proportional odds regression for the primary analysis of the mRS with the sliding dichotomy as a sensitivity analysis. Relative to a conventional dichotomous analysis both of these approaches did indeed lead to substantial efficiency gains, equivalent to more than doubling the sample size.

## Conclusions

SCAST shows that the potential efficiency gains demonstrated in basic methodological research can be realised in practice. This has major implications for the design and analysis of future trials based on ordinal outcome scales.
